# Classification and stratification in pilonidal sinus disease: findings from the PITSTOP cohort

**DOI:** 10.1111/codi.16989

**Published:** 2024-04-21

**Authors:** Matthew J. Lee, Ellen Lee, Mike Bradburn, Daniel Hind, Emily B. Strong, Farhat Din, Arkadiusz P. Wysocki, Jon Lund, Christine Moffatt, Jonathan Morton, Asha Senapati, Helen Jones, Steven R. Brown, the PITSTOP Management Group and the PITSTOP Validators, Ali Khalafalla, Brady Richard, Branagan Graham, Chaudri Sanjay, Di Fabio Francesco, Dennison Godwin, Donnelly David, Evans Martyn, Gerald Francois, Gonzalez Sarah, Grainger Jennie, Hardy Alex, Harilingam Mohan, Hopley Philip, Husain Najam, Kapur Sandeep, Keogh Kenneth, Lim Michael, Mackey Paul, Maeda Yasuko, Mahaptra Sanjay, Mangam Sudhaker, Mazarelo Felix, Muhammad Karim, Pawa Nikhill, Pearce Lyndsay, Pitt James, Rajaganeshan Raj, Shackley Phil, Simmonds Richard, Stevenson Richard, Torkington Jared, Vaughan‐Shaw Peter, Vimalachandran Dale, Wilson Jeremy, Ehsan Aisha, Elsey Elizabeth, Eltyeb Hazim, Harikrishan Athur, Newton Katy, Rabie Mohamed, Williams Annabelle

**Affiliations:** ^1^ Department of Oncology and Metabolism The Medical School, University of Sheffield Sheffield UK; ^2^ Sheffield Clinical Trials Research Unit, School of Health and Related Research (ScHARR) University of Sheffield Sheffield UK; ^3^ Academic Coloproctology Institute of Genetics and Cancer, University of Edinburgh, Western General Hospital Edinburgh UK; ^4^ Logan Hospital Brisbane Australia; ^5^ Royal Derby Hospital University Hospitals of Derby and Burton Derby UK; ^6^ Nottingham University Hospitals NHS Trust Nottingham UK; ^7^ Addenbrookes Hospital Cambridge University Hospitals Cambridge UK; ^8^ St Mark's Hospital London UK; ^9^ Queen Alexandra Hospital Portsmouth UK; ^10^ Oxford University Hospitals NHS Foundation Trust Oxford UK

**Keywords:** classification, pilonidal sinus, proctology, reliability

## Abstract

**Aim:**

Research in pilonidal disease faces several challenges, one of which is consistent and useful disease classification. The International Pilonidal Society (IPS) proposed a four‐part classification in 2017. The aim of this work was to assess the validity and reliability of this tool using data from the PITSTOP cohort study.

**Method:**

Face validity was assessed by mapping the items/domains in the IPS tool against tools identified through a systematic review. Key concepts were defined as those appearing in more than two‐thirds of published tools. Concurrent and predictive validity were assessed by comparing key patient‐reported outcome measures between groups at baseline and at clinic visit. The outcomes of interest were health utility, Cardiff Wound Impact Questionnaire (CWIQ) and pain score between groups. Significance was set at *p* = 0.05 a priori. Interrater reliability was assessed using images captured during the PITSTOP cohort. Ninety images were assessed by six raters (two experts, two general surgeons and two trainees), and classified into IPS type. Interrater reliability was assessed using the unweighted kappa and unweighted Gwet's AC1 statistics.

**Results:**

For face validity items represented in the IPS were common to other classification systems. Concurrent and predictive validity assessment showed differences in health utility and pain between groups at baseline, and for some treatment groups at follow‐up. Assessors agreed the same classification in 38% of participants [chance‐corrected kappa 0.52 (95% CI 0.42–0.61), Gwet's AC1 0.63 (95% CI 0.56–0.69)].

**Conclusion:**

The IPS classification demonstrates key aspects of reliability and validity that would support its implementation.

**What does this paper add to the literature?**

This paper provides a robust assessment of the properties and performance of a tool to classify pilonidal disease based on anatomy and behaviour. It demonstrates face validity and interrater reliability. The tool corresponds with baseline patient‐reported outcome measures (PROMs) but not follow‐up PROMs.

## INTRODUCTION

Classification tools and systems provide surgeons with a framework within which they can describe disease. Many such tools are in use in colorectal conditions such as anal fistula, diverticular disease and piles. Classifying disease using such systems is useful and provides a method of shorthand communication between clinicians, indicating ideas around complexity of disease or anatomy, symptom severity, treatment options and outcomes. It also facilitates comparison of results of studies, with classification systems ensuring that like is compared with like, as well as systematic review and meta‐analysis.

The literature on pilonidal sinus disease (PD) can be criticized for the fact that many researchers make no attempt to classify or stratify the disease [1]. PD can vary from a simple asymptomatic pit to extensive disease with multiple midline pits with lateral extensions, possibly accompanied by marked scarring and deformity from previous sepsis and unsuccessful surgery. Without classification it is difficult to draw meaningful conclusions about comparative studies. There is therefore a pressing need to develop a valid and useful classification system for PD.

Any proposed classification system must be valid and reliable. A valid tool measures what it purports to, and reliability indicates how well a tool measures without error. Many tools have been proposed for PD [2]. However, none of these have been incorporated into mainstream practice. The reasons for this are unclear but may relate to limited data on their clinical utility or awareness of their existence. To date, we are unaware of a comprehensive evaluation of these tools in any PD system. An ideal tool for clinical use would include aspects of face and content validity, i.e. does the tool appear to measure what it claims to, and does it cover all relevant aspects of this description or classification, for example number of pits, sepsis, recurrence, etc [3]? A useful tool should also provide information on symptoms or outcomes (criterion validity including concurrent and predictive) and be reported consistently across users (interrater reliability) [3].

**FIGURE 1:**
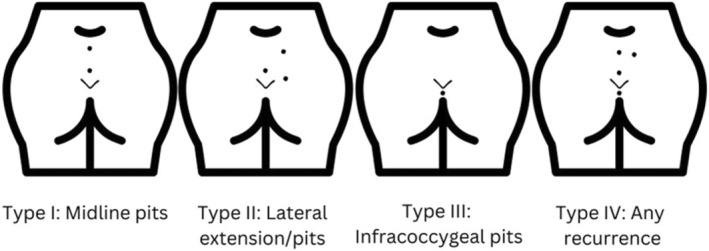
International Pilonidal Society classification schema.

The Pilonidal Sinus: Studying the Options (PITSTOP) study was commissioned by the UK National Institute of Health Research as a first step to improving research on PD. This was a multimethod, multiwork package study based around a prospective cohort study. One of the work packages explored the need to better classify PD. In PITSTOP, the classification tool used was the recently proposed International Pilonidal Sinus Society (IPS) Berlin 2017 classification [4]. This is an expert consensus‐based tool which groups disease into four categories (Figure 1):

- Type 1: only midline pit or sinuses
- Type 2: any midline disease with secondary sinus/es or abscess scar/s
- Type 3: any midline or secondary disease extending below the tip of the coccyx
- Type 4: any disease after treatment with definitive intent.

The aim of this study was to explore (i) face validity, (ii) concurrent and predictive validity and (iii) interrater reliability of the IPS classification system for PD.

## METHOD

### Overview and ethics

PITSTOP was a multicentre prospective cohort study conducted in the UK between 2019 and 2023. It was registered in the ISRCTN registry (95551898). Ethical approval was secured from a NHS Research Ethics Committee prior to commencement (18/EE/0370). Patients gave informed consent to participate in the cohort study (used in the section ‘Concurrent and predictive validity’ below) and for photographs (used in the section ‘Interrater reliability’ below). Clinical participants provided informed consent prior to completion of the exercise in ‘Interrater reliability’.

#### Face validity

Face validity is an assessment of whether a tool measures what it claims to. It is a basic form of assessment and can use a range of qualitative or quantitative approaches [5].

Face validity was established through comparison with eight existing classification systems identified via a previously completed systematic review of classification of PD [2]. The IPS classification was mapped to the concepts assessed in other classification tools synthesized in this systematic review. Important concepts were defined as those reported in more than two‐thirds of the tools (i.e. more than five of the eight existing tools).

#### Concurrent and predictive validity

A tool that demonstrates concurrent validity will demonstrate differences between groups at the time they are classified, for example those with severe disease will have worse quality of life. Predictive validity means that the classification predicts an outcome at a future timepoint after classification, for example quality of life at 6 months postsurgery [3].

Criterion validity was assessed by the ability of the tool to predict key patient outcomes; concurrent validity was assessed using groupings and patient‐reported outcomes at baseline; and predictive validity looked at outcomes at clinic follow‐up. Data were used from the PITSTOP cohort study and grouped according to baseline IPS classification. The PITSTOP cohort was a multicentre prospective cohort study which included all patients who had elective surgery for PD in one of 31 participating UK hospitals. Expecting that treatment selection, especially the degree of intervention, would influence outcomes, analysis of postoperative data was conducted for the groups which had an asymmetric closure procedure, and separately if a skin‐preserving procedure (pit picking, glue, endoscopic pilonidal sinus treatment, Bascom I, laser, seton) had been used. Outcomes of interest were: pain (measured on a visual analogue scale) at baseline and day 7 follow‐up; health utility at baseline and clinic visit measured by crosswalk of values [6] from EQ‐5D‐5L [7]; and the Cardiff Wound Impact Questionnaire (CWIQ) [8] at baseline and clinic visit. Differences between groups were assessed using analysis of variance and significance was set at *p* ≤ 0.05 a priori. Only complete datasets were used for this assessment (i.e. baseline and follow‐up available).

#### Interrater reliability

Interrater reliability of the IPS system was assessed using web‐based photographs of PD taken from patients in the study and classified by groups of surgeons as below.

### Data sources

The image sample set was taken from participants recruited to the PITSTOP cohort study. Participants consented to photography of the surgical site prior to surgery. The operating surgeon graded the disease using the IPS classification system. This has allowed a large database of pictures to be collected, with details of the proposed IPS classification and the type of surgery performed.

It is anticipated that the IPS tool will be used by a range of surgeons if it is shown to meet validity and reliability criteria. Therefore, a maximal variation sampling approach based on experience was taken to recruitment, with the intention to recruit five surgeons to assess photographs for classification of PD from each of the following groups:

- ‘specialists’: defined as those with a high‐volume practice or special interest, and providing a tertiary referral service for PD
- ‘generalists’: those who provide surgery for PD as part of a secondary or general colorectal practice
- ‘trainees’: final year trainees with a declared subspeciality of colorectal surgery.

**FIGURE 2:**
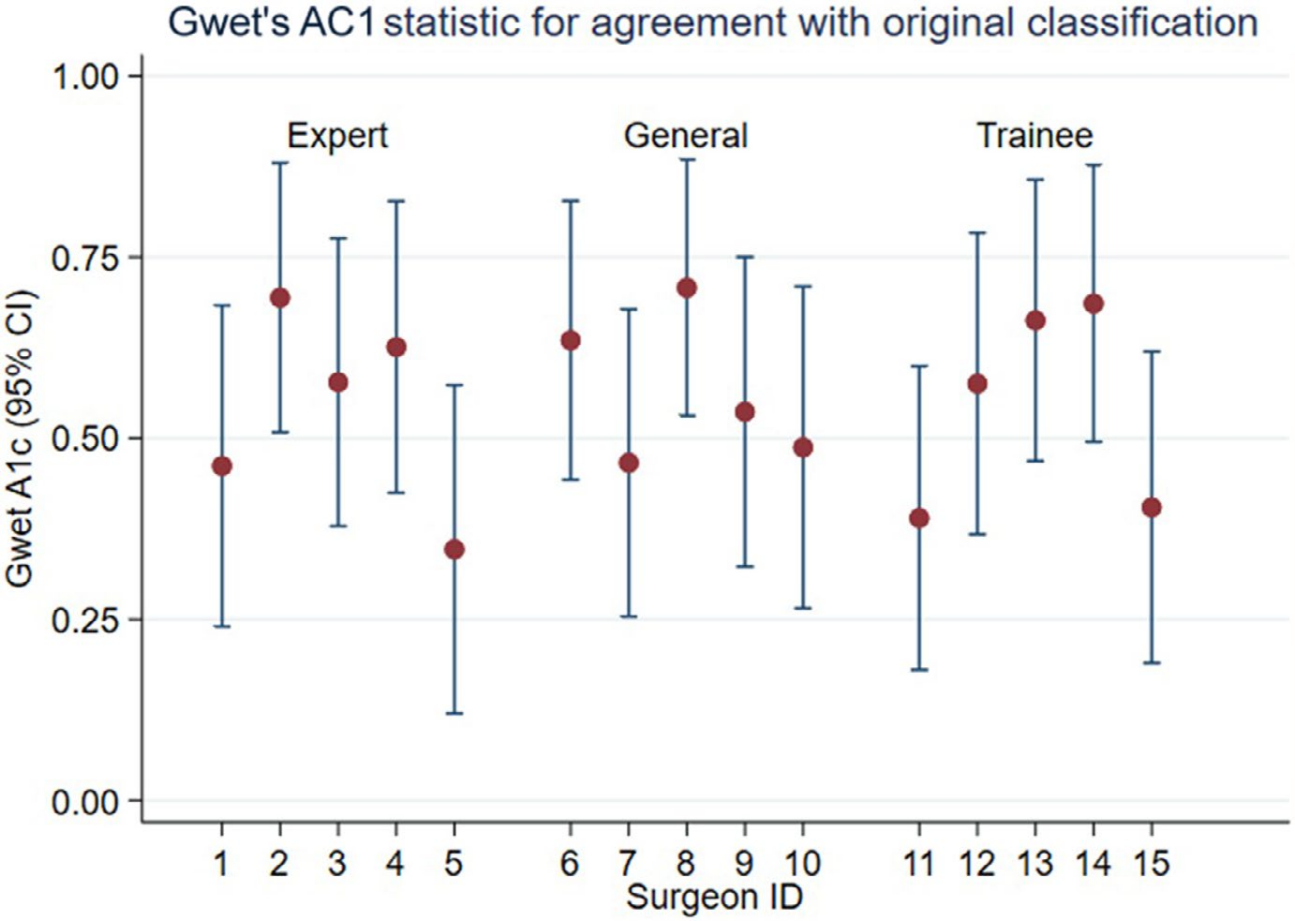
Gwet's AC1 statistic for agreement with original classification.

Each of the 15 assessors was asked to independently rate 36 of the cohort pictures using the IPS classification, with allocation of surgeons to cases selected to ensure overlap with other assessors (Figure 2). A total of 90 photographs were assessed. Each photo was assessed by two ‘specialist’ surgeons, two ‘general’ surgeons and two trainee surgeons. Each assessor was electronically sent the photographs accompanied by a brief medical history (previous PD history including number of elective and emergency procedures). They were not told the classification as recorded by the original surgeon.

Assessors were also asked to record their preferred treatment for each patient they assessed, with the aim being to quantify variation in practice among practitioners. Surgeons recorded their assessments in a Microsoft Excel spreadsheet which was returned to the central trial unit and combined into an analysis dataset. A schema of allocations of photographs is presented in the supplementary material. Assessors did not have to be participants in the PITSTOP cohort study.

### Analysis

Agreement was quantified as both raw and chance‐corrected agreement. Raw agreement is the percentage of patients for whom the assessments agree, whilst chance‐corrected agreement is the ratio of observed to expected agreement. Both raw and chance‐corrected agreement are proportions in which 1 signifies complete agreement whilst 0 reflects complete disagreement. As the four categories are not ordinal, agreement is a simple yes/no construct in which any difference is considered ‘disagreement’. Raw agreement was defined as (100 × number in which all raters agree/number rated) and was accompanied by a 95% Wilson score interval. Chance‐corrected agreement was defined using the unweighted kappa statistic and the unweighted Gwet's AC1 statistics [9]. Agreement among assessors was reported overall and within for ‘specialist’, ‘general’ and ‘trainee’ surgeons. Finally, the agreement was calculated for each assessor in relation to the original assessment made at the time of procedure. Analyses were conducted using Stata version 17.

### Sample size

The sample size was based on (i) a hypothesis test to rule out a minimal kappa statistic, (ii) the standard error of raw agreement and (iii) the number of patients expected to consent and provide usable photographs. For (i), an internal pilot was undertaken in which study surgeons were asked to assess photographs obtained either online or via published articles. A total of 41 pictures were assessed by seven surgeons (five specialists and two trainees) and yielded an overall kappa statistic of 0.42 (0.55 if trainees were excluded). Since the assessments were based on pictures alone and did not include any prior history (e.g. previous surgery), these may be an underestimate. Based on this, a target of kappa = 0.45 was used. The expected lowest limit for chance‐corrected agreement was set at kappa = 0.3, which represents the lowest acceptable agreement if this classification were to be introduced into practice. The sample size calculation also depends on the expected prevalence of each class, which was estimated from the PITSTOP cohort study (approximately 25% type 1, 50% type 2, 10% type 3 and 15% type 4). A sample size of 90 was adequate to rule out differences of 15% between expected and minimum kappa with 90% power and 5% significance; to estimate raw agreement to within a confidence interval half‐width of ±10%; and to be accommodated by the number of photographs available.

## RESULTS

### Face validity

Mapping the items included in the IPS system against eight identified classification systems demonstrated that the IPS reported on key domains of (i) presence of midline pits, (ii) presence of lateral pits, (iii) distant pits (extreme lateral/perianal/lumbar) and (iv) failure of treatment. This indicated that midline pits are featured in seven of the nine systems, lateral pits in seven of the nine systems, distant sinuses in seven of the nine systems and failed treatment/recurrence in eight of the nine systems. Acute presentation and patient factors did not feature in the IPS system, whereas these are addressed in two and four of the nine systems, respectively (Table 1).

**TABLE 1:** Agreement across different systems.

Author	Disease factors				Patient factors	
	Midline pits	Lateral pits	Distant pits	Failed treatment/recurrence/unhealed	Abscess	
Tezel [15]		X	X	X	X	X
Quinodoz et al. [16]	X	X	X			X
Doll [17]	X	X		X	X	
Irkorucu [18]	X	X		X		X
Guner et al. [19]	X	X		X		
Karakas et al. [20]	X	X		X		
Lapsekill et al. [21]				X		
Awad et al. [22]				X		X
IPS	X	X	X	X		
Total reporting	7/9	7/9	3/9	8/9	2/9	4/9

### Criterion and predictive validity

Data from the PITSTOP cohort extracted for patient‐reported outcomes on 667 patients are shown in Table 2. A significant difference was observed in health utility across groups, with type 3 having the lowest health utility at baseline. Pain at baseline was worst in type 4 patients and best in type 2 patients (2.4 vs. 1.7 scored out of 10 on a visual analogue scale). For those who had a skin‐sparing procedure, a significant association was noted between IPS group and CWIQ physical symptoms and stress with quality of life scores worsening across the groups, particularly with type 4 disease. Nonsignificant *p*‐values <0.1 were noted for pain at day 7 (*p* = 0.086) and CWIQ physical symptoms experience (*p* = 0.059). In the asymmetric closure group, no significant differences were noted.

**TABLE 2:** Criterion and predictive validity data for International Pilonidal Society classification.

Baseline	Type 1 (*n* = 179)	Type 2 (*n* = 317)	Type 3 (*n* = 49)	Type 4 (*n* = 99)	*p*
Health utility EQ‐5D‐5L crosswalk	0.8 (0.2)	0.8 (0.2)	0.7 (0.2)	0.8 (0.2)	0.01
Pain at baseline measured on VAS	1.8 (2.3)	1.7 (2.1)	2.2 (2.5)	2.4 (2.4)	0.048

Skin‐sparing procedures	Type 1 (*n* = 102)	Type 2 (*n* = 116)	Type 3 (*n* = 19)	Type 4 (*n* = 31)	*p*
Pain at day 7 measured on VAS	3.0 (2.3)	3.0 (2.2)	3.2 (2.1)	4.0 (2.7)	0.086
Health utility at clinic follow‐up EQ‐5D‐5L crosswalk	0.8 (0.2)	0.8 (0.2)	0.7 (0.2)	0.8 (0.2)	0.138
CWIQ physical symptoms and daily living experience at clinic	93.9 (10.4)	92.7 (13.9)	89.8 (15.9)	84.6 (25.3)	0.059
CWIQ physical symptoms and daily living stress at clinic visit	97.2 (6.0)	95.5 (12.0)	91.4 (16.1)	88.8 (23.8)	0.03
CWIQ QoL at clinic visit	8.7 (1.3)	8.7 (1.6)	8.9 (1.0)	8.0 (2.5)	0.194
CWIQ satisfaction at clinic visit	8.6 (1.5)	8.8 (1.7)	8.8 (1.0)	8.0 (2.3)	0.246
CWIQ well‐being at clinic visit	70.3 (21.9)	69.6 (21.6)	70.8 (20.1)	58.8 (28.3)	0.167

Asymmetric closure	Type 1 (*n* = 46)	Type 2 (*n* = 148)	Type 3 (*n* = 20)	Type 4 (*n* = 54)	*p*
Pain at day 7 measured on VAS	1.8 (2.0)	1.8 (2.2)	1.6 (1.9)	2.3 (2.9)	0.719
Health utility at clinic follow‐up EQ‐5D‐5L crosswalk	0.9 (0.1)	0.9 (0.2)	0.9 (0.2)	0.9 (0.2)	0.43
CWIQ physical symptoms and daily living experience at clinic	85.9 (20.3)	82.4 (19.8)	73.2 (29.1)	78.3 (25.1)	0.181
CWIQ physical symptoms and daily living stress at clinic visit	88.3 (21.7)	86.9 (19.0)	75.0 (34.0)	82.2 (24.3)	0.126
CWIQ QoL at clinic visit	7.7 (1.7)	7.7 (1.9)	6.8 (2.8)	7.1 (2.3)	0.168
CWIQ satisfaction at clinic visit	7.7 (1.9)	7.6 (2.0)	6.4 (2.8)	7.0 (2.4)	0.704
CWIQ well‐being at clinic visit	58.2 (24.3)	59.9 (21.3)	54.2 (27.7)	53.3 (26.2)	0.392

*Note*: Pain (measured on a visual analogue scale) at baseline and day 7 follow‐up, health utility at baseline and clinic visit, and the CWIQ at baseline and clinic visit. All values are given as mean (SD).

Abbreviations: CWIQ, Cardiff Wound Impact Questionnaire; QoL, quality of life; VAS, visual analogue scale.

### Interrater reliability

The initial classification of case photographs and their associated operations is presented in Table 3. Raters for all three groups were recruited as planned. The agreement among surgeons is summarized in Table 3. Of the 540 assessments (90 patient photographs and case histories each having six assessments), 14 (3%) of assessments were classified as ‘none of the above’ affecting 12 (13%) of the included patients.

**TABLE 3:** Interrater agreement.

Surgeon	No. in agreement^a^	Percentage (95% CI)	Kappa (95% CI)	Gwet's AC1 (95% CI)
Specialist	65	72% (62%–80%)	0.54 (0.38–0.69)	0.67 (0.56–0.79)
General	63	70% (60%–78%)	0.54 (0.40–0.69)	0.64 (0.52–0.76)
Trainee	64	71% (61%–79%)	0.58 (0.43–0.72)	0.65 (0.54–0.77)
Overall	34	38% (28%–48%)	0.52 (0.42–0.61)	0.63 (0.56–0.69)

^a^ Number in agreement is the number where both assessors agree (or all six assessors for overall agreement). 95% confidence intervals are used throughout.

Overall, the six assessors all reached the same consensus in 38% of participants with a chance‐corrected kappa statistic of 0.52 (95% CI 0.42–0.61) and a Gwet's AC1 statistic of 0.63 (95% CI 0.56–0.69). Agreement between pairs was higher with ‘specialist’ surgeons agreeing in 72% of patients, ‘general’ surgeons agreeing in 70% of patients and ‘trainee’ surgeons agreeing in 71% of patients. The overall agreement is lower since this measure required all six assessors to agree. All six surgeons agreed in 34 (38%) of patients, and at least five of the six surgeons agreed in 53 (59%). The chance‐corrected kappa agreement was above 0.5 (conventionally considered ‘moderate’) and the Gwet's AC1 measure was over 0.6 for all subgroups of surgical expertise.

Assessors were less likely to agree with the original classification than with other assessors when given the same photograph. Raw agreement ranged between 47% (17/36) and 75% (27/36) with chance‐corrected kappa statistics ranging from 0.11 to 0.59, and chance‐corrected Gwet's AC1 agreement statistics of 0.35–0.71 (Figure 2).

**FIGURE 3:**
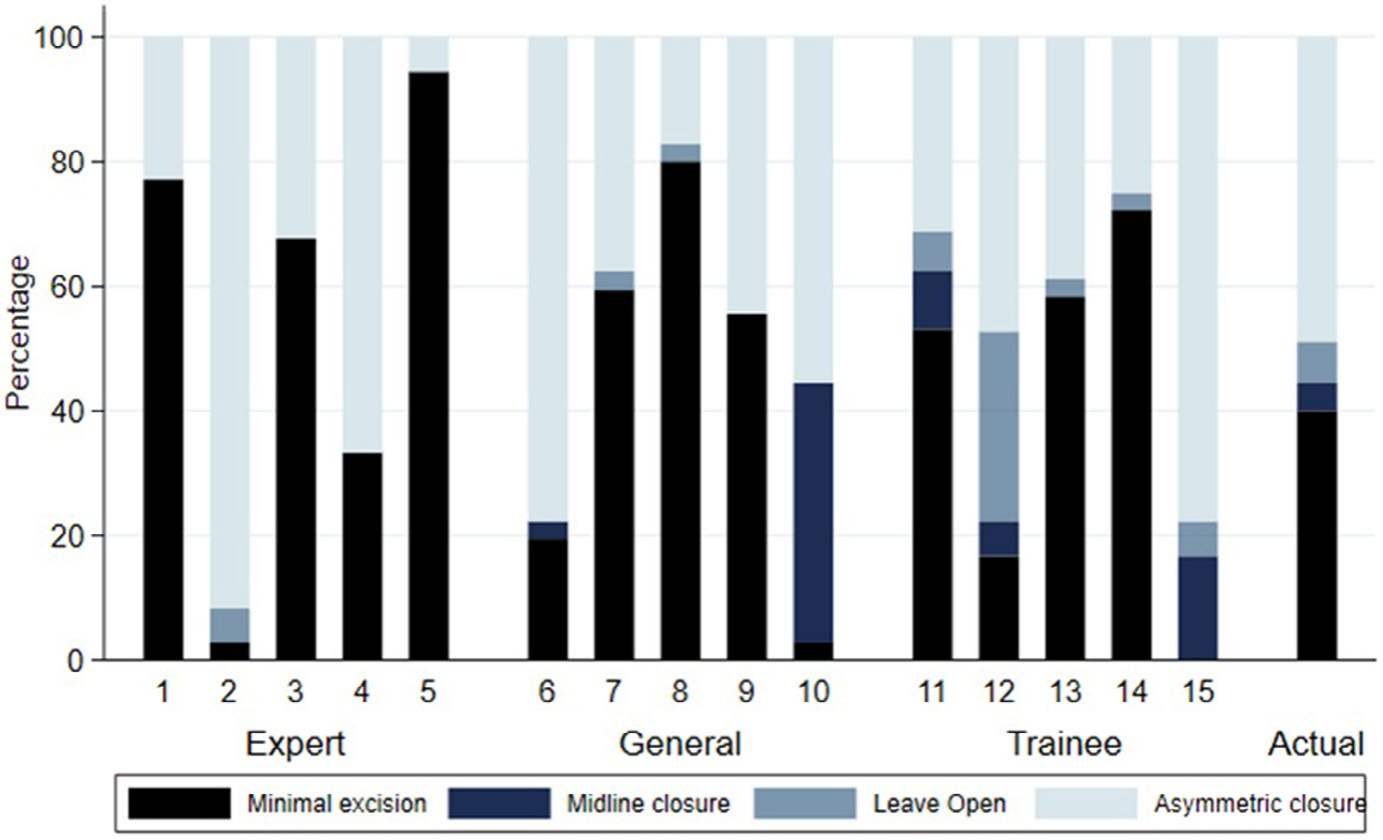
Treatment proposed by raters for each case.

The surgeons surveyed were slightly more likely to recommend minimally invasive surgery (46%) than those who actually received this approach (40%). Although the percentage favouring asymmetric closure (45%) was similar to the treatment actually received (49%) the specific procedure types differed: the surgeons surveyed were more likely to use a Bascom cleft lift (22%) than a Karydakis (16%). The use of midline closure (5%) and leave open (4%) approaches were uncommon, most notably among the specialist surgeons surveyed (Figure 3).

## DISCUSSION

This paper has explored the validity and reliability of the IPS classification, demonstrating favourable characteristics to support its use in practice. The assessment of face validity shows that the concepts assessed in the tool overlap with those assessed elsewhere. The IPS tool shows criterion validity, with different groups having different ratings of pain and health utility at baseline. The predictive validity of the tool appears less strong, with only the skin‐preserving procedure group demonstrating significant univariate associations between baseline classification and two patient‐reported outcomes. This association was not seen in the asymmetric closure group. Finally, the interrater reliability of the tool shows good agreement between raters, suggesting this is a reproducible classification.

The tool seems to address key aspects of face validity, with the common areas of disease anatomy included in the dataset. Aspects such as acute disease and patient factors are seemingly infrequently reported in tools and therefore omission from this system seems appropriate. It is possible to draw parallels from this system to other disease classification systems. This includes Parks classification of anal fistula, which is based on fistula tract in relation to the anal sphincter [10]. The Hinchey system is another commonly used disease classification system [11]; it categorizes complicated diverticular disease into four categories. Neither of these systems distinguishes on the basis of patient factors.

One possible explanation for limited predictive validity of the IPS is the lack of consistency in treatment selection according to class of disease. This is reflected both in the data presented in Figure 3, which shows broad variation in selection across surgeons, and in a recently published survey [12]. There were some differences between groups in the skin‐sparing procedure subgroup but not in the asymmetric closure group. This suggests that the impact of treatment may override the impacts of initial disease severity in the latter group. Given the potential variable impacts of treatments on different disease types, it is conceivable that this heterogeneity masks any true effects. However, the tool does demonstrate qualities of concurrent validity, with patients in different classes reporting different pain and health utility scores prior to intervention.

It is interesting to note aspects of agreement within the interrater reliability study, specifically that raters were more likely to agree with each other in the exercise than the original rater. This is despite images of the PD as well as relevant history. It is not clear if the clinician assessing a patient face to face picked up additional data to change the classification. The moderate levels of interrater agreement do suggest that the tool may offer a means to succinctly summarize disease type between surgeons. The magnitude of reliability certainly exceeds previously reported and often used classifications such as grading of haemorrhoids (kappa 0.38) [13], and dysplastic colorectal adenomas (kappa 0.38) [14]. One limitation of the IPS is the fact that 3% of the images were not able to be classified. It is also interesting to note that there was a difference in classification between trainees and others. It is not clear why this arose. While the rules of the system should be easy to apply, it might be that some subtleties are not clear to those with less experience of the condition. This requires further investigation if the tool is widely adopted.

The study is not without limitations. The assessment of concurrent and predictive validity used only univariate analyses and may have provided false negative results on associations. Some of the types of PD (i.e. type 3) were uncommon in the cohort and therefore less well represented here. While unlikely due to the robust approaches used here, there is a possibility this may impact the findings of this study. The variation between baseline assessment and remote raters in the interrater reliability study suggest that photographs may not provide all the relevant information. This does have an impact on rating of agreement between baseline assessors and the two independent assessors in this study. However, the strength of agreement between the two independent raters does reassure us about the reproducibility of the system. The PITSTOP cohort was open to all patients undergoing elective surgery for PD at each participating centre. There was no selection based on disease characteristics. It is therefore unlikely that selection bias has influenced our results.

The strengths of this study include an assessment of different aspects of reliability and validity using different methods but anchored on a robust dataset. It has also tested reproducibility across surgeons with different levels of experience. The study also provides data on the relationship of the classification on patient‐reported outcomes.

The data from this study provide insight into the clinical relevance of the IPS classification system and suggest that this might be useful as a risk stratifier in clinical audit. We propose that inclusion of this system should be used in future research to allow data to be more easily compared between studies. Future trials may wish to stratify randomizations by disease classification to allow isolation of the effects of treatments on different classifications.

The IPS tool shows moderate interrater reliability, appears to have content validity and demonstrates concurrent validity. This means it may be a helpful tool in practice; therefore clinicians and policy makers should consider routine use in practice.

## AUTHOR CONTRIBUTIONS

**Matthew J. Lee:** Conceptualization; funding acquisition; writing – original draft; writing – review and editing; visualization; methodology; investigation. **Ellen Lee:** Data curation; formal analysis; methodology; project administration; validation; visualization; writing – original draft; writing – review and editing. **Mike Bradburn:** Data curation; formal analysis; methodology; project administration; funding acquisition; writing – original draft; writing – review and editing; visualization; validation. **Daniel Hind:** Conceptualization; funding acquisition; methodology; project administration; resources; software; writing – original draft; writing – review and editing. **Emily B. Strong:** Project administration; data curation; writing – review and editing; writing – original draft. **Farhat Din:** Conceptualization; funding acquisition; investigation; writing – review and editing. **Arkadiusz P. Wysocki:** Investigation; writing – review and editing. **Jon Lund:** Conceptualization; investigation; funding acquisition; writing – review and editing. **Christine Moffatt:** Conceptualization; funding acquisition; writing – review and editing. **Jonathan Morton:** Conceptualization; investigation; funding acquisition; writing – review and editing. **Asha Senapati:** Conceptualization; funding acquisition; investigation; writing – review and editing. **Helen Jones:** Writing – review and editing; investigation. **Steven R. Brown:** Conceptualization; funding acquisition; writing – original draft; investigation; visualization; writing – review and editing.

## FUNDING INFORMATION

This study was funded by the NiHR Research grant HTA 17/17/02.

## CONFLICT OF INTEREST STATEMENT

All authors have completed the unified competing interest form at www.icmje.org/coi_disclosure.pdf (available on request from the corresponding author) and declare: (1) no financial support for the submitted work from anyone other than their employer; (2) no financial relationships with commercial entities that might have an interest in the submitted work; (3) no spouses, partners or children with relationships with commercial entities that might have an interest in the submitted work; and (4) no nonfinancial interests that may be relevant to the submitted work. Mike Bradburn is a current member of the HTA Commissioning Committee. Steven Brown was a member of HTA Commissioning Committee October 2017 to September 2019. Daniel Hind was a member of the HTA Clinical Evaluation and Trials Committee and HTA Fast Track Committee – June 2021.

## ETHICS STATEMENT

Ethical approval was secured from a National Health Service (NHS) Research Ethics Committee before commencement (18/EE/0370).

## Supporting information


Figure S1:

## Data Availability

Data are available on request from the Sheffield Clinical Trials Research Unit, School of Health, and Related Research (ScHARR), University of Sheffield, Sheffield, UK.

## APPENDIX A

### The PITSTOP Management Group

Ali Khalafalla, Brady Richard, Branagan Graham, Chaudri Sanjay, Di Fabio Francesco, Dennison Godwin, Donnelly David, Evans Martyn, Gerald Francois, Gonzalez Sarah, Grainger Jennie, Hardy Alex, Harilingam Mohan, Hopley Philip, Husain Najam, Kapur Sandeep, Keogh Kenneth, Lim Michael, Mackey Paul, Maeda Yasuko, Mahaptra Sanjay, Mangam Sudhaker, Mazarelo Felix, Muhammad Karim, Pawa Nikhill, Pearce Lyndsay, Pitt James, Rajaganeshan Raj, Shackley Phil, Simmonds Richard, Stevenson Richard, Torkington Jared, Vaughan‐Shaw Peter, Vimalachandran Dale, Wilson Jeremy.

### The PITSTOP Validators

Ehsan Aisha, Elsey Elizabeth, Eltyeb Hazim, Harikrishan Athur, Newton Katy, Rabie Mohamed, Williams Annabelle.
