# Supplementary material for: Classification and stratification in pilonidal sinus disease: findings from the PITSTOP cohort
Source: Colorectal Dis. 2024 Apr 21;27(1):e16989. doi: 10.1111/codi.16989 (PMC11683182; doi:10.1111/codi.16989)
Supplement: Supplementary file 1 — Figure S1: [file CODI-27-0-s001.docx]

Supplementary Figure: Allocation of patients with PD to surgical assessors

| **Patient ID** | **Assessor ID** | | | | | **Surgical assessor** |
| --- | --- | --- | --- | --- | --- | --- |
|  | E1 | E2 | E3 | E4 | E5 |  |
|  | G6 | G7 | G8 | G9 | G10 |  |
|  | T11 | T12 | T13 | T14 | T15 |  |
| 1 to 9 |  |  |  |  |  | 1&2, 6&7, 11&12 |
| 10 to 18 |  |  |  |  |  | 1&3, 6&8, 11&13 |
| 19 to 27 |  |  |  |  |  | 1&4, 6&9, 11&14 |
| 28 to 36 |  |  |  |  |  | 1&5, 6&10, 11&15 |
| 37 to 45 |  |  |  |  |  | 2&3, 7&8, 12&13 |
| 46 to 54 |  |  |  |  |  | 2&4, 7&9, 12&14 |
| 55 to 63 |  |  |  |  |  | 2&5, 7&10, 12&15 |
| 64 to 72 |  |  |  |  |  | 3&4, 8&9, 13&14 |
| 73 to 81 |  |  |  |  |  | 3&5, 8&10, 13&15 |
| 82 to 90 |  |  |  |  |  | 4&5, 9&10, 14&15 |
